# Phosphodiesterase inhibitors as adjunctive therapies for tuberculosis

**DOI:** 10.1016/j.ebiom.2016.02.016

**Published:** 2016-02-09

**Authors:** Bintou Ahmadou Ahidjo, William R. Bishai

**Affiliations:** a Center for Tuberculosis Research, Johns Hopkins University School of Medicine, Baltimore, MD, USA; b Howard Hughes Medical Institute, Chevy Chase, MD, USA

*Mycobacterium tuberculosis* (Mtb) is one of the most successful human pathogens, and a key mechanism in its virulence arsenal is its ability to influence host immune responses to its advantage. In recent years it has become clear that one such mechanism pivots on bacterial-derived, secreted small molecules that are released from the intracellular phagosome of macrophages into the host cell cytosol where they interfere with host cell signaling and downstream cytokine responses. Using small molecules such as cyclic nucleotides, Mtb subverts the host's innate bactericidal responses to remodel the intracellular niche to an environment favorable for mycobacterial survival or growth (Agarwal et al., 2009; Dey et al., 2015; Tobin et al., 2012; Wallis and Hafner, 2015).

A key bacterial-derived, secreted small molecule is the well-known second messenger cyclic adenosine monophosphate (cAMP). Upon infection Mtb produces a burst of cAMP within macrophages. Through a microbial adenylate cyclase gene, bacterial-derived cAMP is delivered to the macrophage cytoplasm increasing cytosolic cAMP levels 3–5-fold above baseline and triggering the PKA-CREB pathway to upregulate NFκB transcription. One consequence of bacterial subversion of host cAMP signaling is the elevated TNF-α secretion at the early stages of infection promoting necrosis and granuloma formation—outcomes that foster bacterial survival (Agarwal et al., 2009).

Mtb also interferes with immune signaling by secreting another bacterial-derived second messenger, cyclic-di-adenosine monophosphate (c-di-AMP) (Dey et al., 2015). This pathogen-associated molecular pattern (PAMP) which is recognized by the macrophage cytosolic surveillance pathway behaves as a double-edged sword in Mtb pathogenesis. On the one hand, it contributes to the induction of Type I interferon levels through the STING-IRF3 signaling pathway, enhancing immunopathology and thus benefiting the microbe. On the other hand, c-di-AMP also enhances autophagy and bacterial killing. Mtb expressing excess c-di-AMP displays a loss of pathogenicity in animal models indicating that the dominant impact of microbial c-di-AMP production is its stimulation of autophagy to benefit the host (Dey et al., 2015). This latter observation suggests that measures to prevent the breakdown of Mtb-derived c-di-AMP might be beneficial for host control of tuberculosis (TB).

The failure to control the global TB epidemic despite the availability of curative drug regimens is partly driven by the inherent difficulties of maintaining continuous chemotherapy over at least six months (WHO, 2015). Moreover, even when patients are cured from the disease, lung function is often never fully recovered. As such, adjunctive host-directed therapies (HDTs) for TB are currently being explored to improve treatment outcomes by restoring effective host immunity, achieving an appropriate degree of inflammation, and preventing disease-associated lung pathology (Wallis and Hafner, 2015). Success in modulating immunity may also lead to treatment shortening by reducing granulomatous pathology and the bacterial persister-state associated with granulomas.

Small molecule phosphodiesterase (PDE) inhibitors – which raise levels of certain cytosolic cyclic nucleotides – have become important drugs in human medicine with the introduction of PDE3 inhibitors for intermittent claudication, PDE5 inhibitors for erectile dysfunction and pulmonary hypertension, and PDE4 inhibitors for chronic obstructive pulmonary disease. PDE4 inhibitors have been of particular interest for lung infections since they reduce pulmonary inflammation. Not surprisingly, the evaluation of FDA-approved human PDE inhibitors as well as those in the pipeline for FDA approval has emerged as an attractive strategy for adjunctive HDTs against TB.

PDE inhibitors are isoenzyme-specific compounds of different binding affinities and potencies that also act according to the tissue distribution of the isozyme (Wang and Cui, 2006). Several PDE inhibitors have already shown varying degrees of success as adjunctive TB treatment agents (Maiga et al., 2013, Maiga et al., 2015; Subbian et al., 2011). Addition of an experimental PDE4 inhibitor—rolipram—to standard TB therapy in the mouse model, for example, had no impact on the rate of bacterial clearance at six months (Maiga et al., 2013). However, more recently roflumilast, an FDA approved PDE4 inhibitor was shown to augment the action of isoniazid in an 8-week mouse model (Maiga et al., 2015). Furthermore, other PDE classes have also shown benefit. Addition of the PDE3 inhibitor cilostazol or the PDE5 inhibitor sildenafil reduced bacterial clearance and accelerated the time-to-tissue sterilization by up to one month when added to the full 6-month standard regimen in a mouse model (Maiga et al., 2013).

In this issue, Subbian et al. assess the adjunctive value of the PDE inhibitor CC-11050 when used in combination with isoniazid to treat TB (Subbian et al., 2016). CC-11050, which is currently in clinical trials for other indications, is a new PDE4 inhibitor. Using the rabbit model of TB, Subbian and colleagues showed that adjunctive use of CC-11050 with isoniazid results in a significant reduction of pulmonary bacillary burden. They further demonstrated that the drug dampens the TNF-α regulatory network, reduces macrophage activation and the lung inflammatory responses, and it lessens pulmonary fibrosis and necrosis (Subbian et al., 2016). This CC-11050-specific effect is attributable to its host modulatory properties as it had no antibacterial activity or apparent drug–drug interactions with isoniazid. These data demonstrate that CC-11050 has activity as an adjunctive HDT and could potentially shorten the duration of TB treatment (Subbian et al., 2016). As with CC-3052 and roflumilast, the beneficial effect of CC-11050 was observed at approximately 8-weeks post-treatment (Maiga et al., 2015; Subbian et al., 2011, Subbian et al., 2016). While it remains unknown how CC-11050 will perform beyond this time period, the reduced inflammatory response and associated increased bactericidal activity of isoniazid may result in fewer bacilli entering persister-state. It is also tempting to speculate that the reduced fibrosis observed may lead to alterations in granuloma structure allowing for greater antibiotic penetration. It will be important to assess CC-11050 with a full multidrug therapy and accompanying relapse studies to determine if the 8-week study combining CC-11050 with a single antibiotic will translate into a benefit when the drug is added to a 6-month multidrug therapy. Nonetheless, given the explosive growth of immunomodulatory drugs entering clinical use for inflammatory diseases, these findings continue to offer room for optimism that TB-dysregulated immunity may be corrected pharmacologically by HDTs. The challenge will be to determine the right cocktail and timing of these potent immunomodulators with tried-and-true multidrug antibiotic therapy.

## Disclosure

The authors have no relevant conflicts of interest to disclose.
